# Spectroscopic characterization of photoaccumulated radical anions: a litmus test to evaluate the efficiency of photoinduced electron transfer (PET) processes

**DOI:** 10.3762/bjoc.9.91

**Published:** 2013-04-24

**Authors:** Maurizio Fagnoni, Stefano Protti, Davide Ravelli, Angelo Albini

**Affiliations:** 1 PhotoGreen Lab, Department of Chemistry, University of Pavia, V. Le Taramelli 12, 27100 Pavia, Italy

**Keywords:** aromatic nitriles, persistent radical anion, photochemical activation, photoinduced electron transfer (PET), photooxidant, reactive intermediates

## Abstract

Steady-state irradiation in neat acetonitrile of some aromatic nitriles, imides and esters (10^−5^–10^−3^ M solution) in the presence of tertiary amines allowed the accumulation of the corresponding radical anions, up to quantitative yield for polysubstituted benzenes and partially with naphthalene and anthracene derivatives. The condition for such an accumulation was that the donor radical cation underwent further evolution that precluded back electron transfer and any chemical reaction with the radical anion. In fact, no accumulation occurred with 1,4-diazabicyclo[2.2.2]octane (DABCO), for which this condition is not possible. The radical anions were produced from benzene polyesters too, but decomposition began early. Ipso substitution was one of the paths with secondary amines and the only reaction with tetrabutylstannane. The results fully support the previously proposed mechanism for electron transfer (ET) mediated photochemical alkylation of aromatic acceptors via radical ions and radical intermediates.

## Introduction

**Scheme 1:**
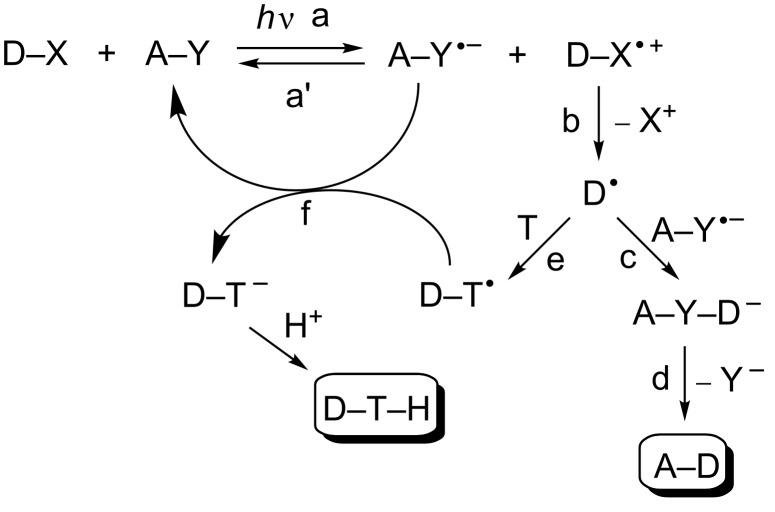
Photoinduced electron transfer as an access to radical chemistry.

Redox reactions between organic molecules have a limited scope because of the rarely matched redox potential. On the other hand, the very structure of electronically excited states makes them both easily oxidized (by donating the electron promoted to an empty orbital) and reduced (an electron is transferred to the low-lying semi-occupied orbital). As a result, redox reactions are quite common in photochemistry and occur under mild conditions, avoiding the use of aggressive inorganic reagents otherwise required. Photoinduced electron transfer indeed offers an advantageous access to radical ions as well as the possibility to control the ensuing chemistry, in a way hardly attained through electrochemical or chemical methods [1–6]. A radical ion formed at a cathode/anode finds itself in an environment where electrons/holes are abundant. Likewise, reducing/oxidizing chemicals must be used at a sufficiently high concentration to be active, and again the radical ions are formed in an environment where a subsequent electron-transfer step in the same direction is likely. In contrast, photoinduced electron transfer generates a radical-ion pair. Electron transfer in the reverse direction (back electron transfer, BET) is thus likely and leads again to the starting molecules [7], unless one or both of the radical ions undergoes a sufficiently fast reaction (Scheme 1). In the simplest case, one of the radical ions reacts, while the other one persists. At some point in the mechanism, BET occurs, such that the final product has undergone no net change of the oxidation level, or an equimolecular mixture of a reduced and an oxidized product is formed.

As an example, the largely positive reduction potential of aromatic nitriles [5,8–9] and cyanophthalimides [10] in the singlet excited state or of aromatic esters in the triplet state [5,11] makes PET a common occurrence upon irradiation of such substrates in the presence of a variety of donors (D–X, step a). In all of the above cases, the generated radical anion (A–Y^•−^) is a persistent species easily detected by laser flash photolysis. This approach applies to a variety of donors, including amines [12–13], carboxylic acids or their derivatives [14–15], aliphatic acetals and ketals [16], ethers [17], organostannanes [18–19], organosilanes [20–22], aromatics [10,23–24], and even alkanes [25–27]. BET (path a') could ensue, thus leading to no chemical change. However, when the D–X^•+^ intermediate contained a good electrofugal group (such as a silyl, stannyl, *t*-Bu group or a hydrogen [12–27]), unimolecular fragmentation was possible and gave neutral alkyl radicals (D^•^) with a reasonable (0.1 or higher) quantum yield (Scheme 1, step b) [5]. Addition to the aromatic radical anion and re-aromatization gave an alkylated aromatic (A–D, path c → d), while trapping of the radical by an electron-withdrawing substituted alkene T (step e) [28–29] followed by BET from the radical anion (step f) of the acceptor (that was thus recovered) led to photocatalyzed alkylation of the alkene [5,30–31]. Both processes have found some application in synthesis for the mild and selective activation of aliphatic derivatives, in particular of a C–H bond [32].

The role of the acceptor radical anion in the above processes is thus decisive, and we report below a steady-state investigation of such species arising from aromatic compounds known to participate in photosubstitution or photocatalytic alkylation reactions. It was proposed to ascertain whether these may accumulate, as often observed in electrochemistry [33–34] and by pulse radiolysis [35], but rarely in photochemistry.

## Results

**Figure 1:**
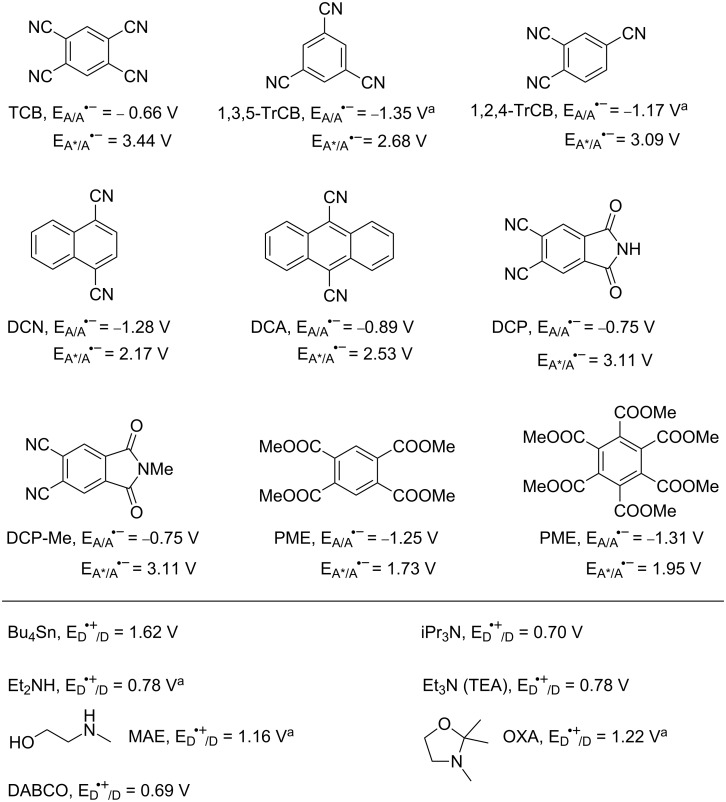
Reduction potential (versus SCE) of the ground and excited state of acceptors and oxidation potential of the ground-state donors examined in this work. ^a^Value measured in the present work (see Experimental).

The acceptors chosen for this study were nitriles, viz. 1,2,4,5-tetracyanobenzene (TCB), 1,3,5-tricyanobenzene (1,3,5-TrCB), 1,2,4-tricyanobenzene (1,2,4-TrCB), 1,4-dicyanonaphthalene (DCN), 9,10-dicyanoanthracene (DCA); imides, viz. 4,5-dicyanophthalimide (DCP), and its *N*-methylated derivative (DCP-Me); and esters (methyl pyromellitate, PME, and methyl mellitate, ME). Radical anions of this type have been previously observed in glasses (e.g., from the irradiation of TCB and α-methylstyrene in isoamyl alcohol glass at 113 K) [36] and in a few cases in fluid solution (from dicyanoanthracene in the presence of methoxide [37] and from 2,6,9,10-tetracyanoanthracene with amines [38]). As for the donors, these were chosen on the basis of their oxidation potential (see Table 1 and Figure 1), to allow for an overall exergonic electron transfer from the donor to the excited acceptor in all of the cases considered below [39]. The relevant redox parameters for the ground states of acceptors and donors are gathered in Figure 1, along with the reduction potentials for the excited states of the aromatics.

**Table 1:** Spectroscopic properties of the examined radical anions.

acceptor/ donor	A^•−^ (main band) λ_max_ (nm)	ε (× 10^−3^) L mol^−1^ cm^−1^	A^•−^ (further maxima) λ_max_ (nm)	Φ_A_^•−^

TCB/OXA	462	10.3	436, 414, 375, 354	0.15
TCB/TEA		9.6		0.15
TCB/iPr_3_N		9.8		0.08
TCB/Et_2_NH^a^		6.7		0.15
TCB/MAE^a^		2.9		0.05
TCB (literature)^b,c^	462	4.9,^b^ 14.6–15.4^c^	–	–
1,2,4-TrCN/OXA	351	2.3	433, 397	–
DCN/OXA	389, 512	5.4, 1.0	621, 500, 481	–
DCN (literature)^d^	512	3.6^d^	–	–
DCA/OXA	705, 640	0.6, 0.4	520	–
DCA (literature)^e^	640	5.6^e^	–	–
DCP/OXA	577	25.6	536, 382	–
DCP-Me/OXA	578	26.3	537, 390	–
ME/OXA^a^	527	0.80	368	–
PME/OXA^a^	533	1.4	371	–

^a^An irreversible reaction occurred. ^b^Measured by pulse radiolysis in 2-methyltetrahydrofuran, at 77 K; from [35]. ^c^Measured by laser flash photolysis at room temperature of a MeCN solution of 1,2,4-trimethylbenzene or *o*-xylene as donors, see [24]. ^d^Measured by laser flash photolysis at room temperature of a DMF solution of alkyltriphenyl borate anions as donors; from [40]. ^e^Measured by electrolysis in DMF at room temperature; from [34].

**Figure 2:**
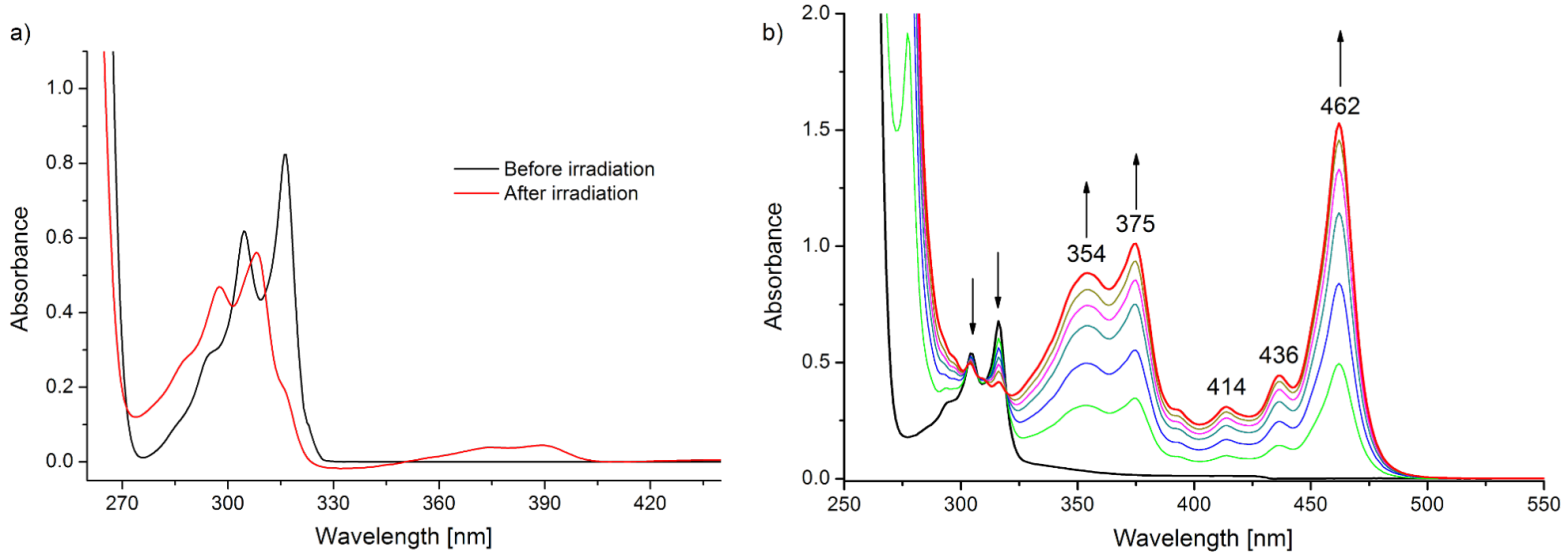
UV-monitoring of: (a) a 2 × 10^−4^ M solution of TCB in the presence of Bu_4_Sn (10^−2^ M) and (b) a 1.5 × 10^−4^ M solution of TCB in the presence of OXA (5 × 10^−2^ M) in freeze–pump–thaw deoxygenated MeCN (λ_IRR_ = 313 nm) from 0 (black line) to 15 min (red line).

In the experiments, a MeCN solution of the acceptor in the presence of the chosen donor was deaerated by freeze–pump–thaw technique and irradiated at 313 nm. Thus, irradiation of a 2 × 10^−4^ M solution of TCB in the presence of Bu_4_Sn (10^−2^ M) caused a stepwise blue shift of the near UV band of TCB and a weak absorption in the visible (see Figure 2a), along with a fluorescence peak centered at 510 nm. The conversion was complete after a few minutes, and readmitting air caused little change in the spectrum. Chromatographic examination confirmed the complete consumption of TCB and the formation of a single product, identified as 5-butyl-1,2,4-tricyanobenzene by comparison with an authentic sample [18]. The measured quantum yield of TCB consumption was 0.40. The examination was then extended to a series of nitrogen-based donors. In the presence of 2,2,3-trimethyloxazolidine (OXA), a yellow color developed and a spectrum with maxima at 462, 375 and 354 nm was registered (see Figure 2b) along with shoulders at 436 and 414 nm. The final absorption was qualitatively and quantitatively close to that reported in the literature for the TCB radical anion (see Table 1). Thus, conversion was deemed to be complete, with a 0.15 quantum yield of formation of TCB^•−^. Readmitting air in this case caused the disappearance of the color and the full regeneration of the starting nitrile, as confirmed by chromatographic analysis.

Exactly the same behavior was observed by using triethylamine (TEA) as the donor. It is noteworthy from the practical point of view that with OXA the TCB^•−^ was formed at about the same rate also in a nitrogen- or argon-flushed solution, whereas with TEA and the other donors discussed below a more rigorous deaeration such as the freeze–pump–thaw method was required. Again similar was the behavior of triisopropylamine, which showed a somewhat lower quantum yield (0.08), however, in the formation of TCB^•−^. The use of diethylamine (Et_2_NH) led initially to the accumulation of the TCB^•−^ absorption up to ca*.* 2/3rd of the maximum value with TEA, but then a different evolution began to manifest. In this case, oxygen quenching eliminated the radical anion but left some absorption (with maxima at 390 and 290 nm). With *N*-(methylamino)ethanol (MAE) a lesser amount of TCB^•−^ was formed and the new absorption at 290 nm, as above, was apparent already during the irradiation (not shown). On the other hand, the TCB^•−^ spectrum did not develop in the presence of DABCO. In this case, TCB was only sluggishly consumed, and a new absorption band with a maximum at 380 nm grew, which was unaffected when air was readmitted.

The investigation was then extended to further acceptors by using TEA and OXA, which had demonstrated to be the best donors for the accumulation of the acceptors radical anions. Actually, almost superimposable results were obtained, and in the following, only the results with the latter donor are mentioned.

**Figure 3:**
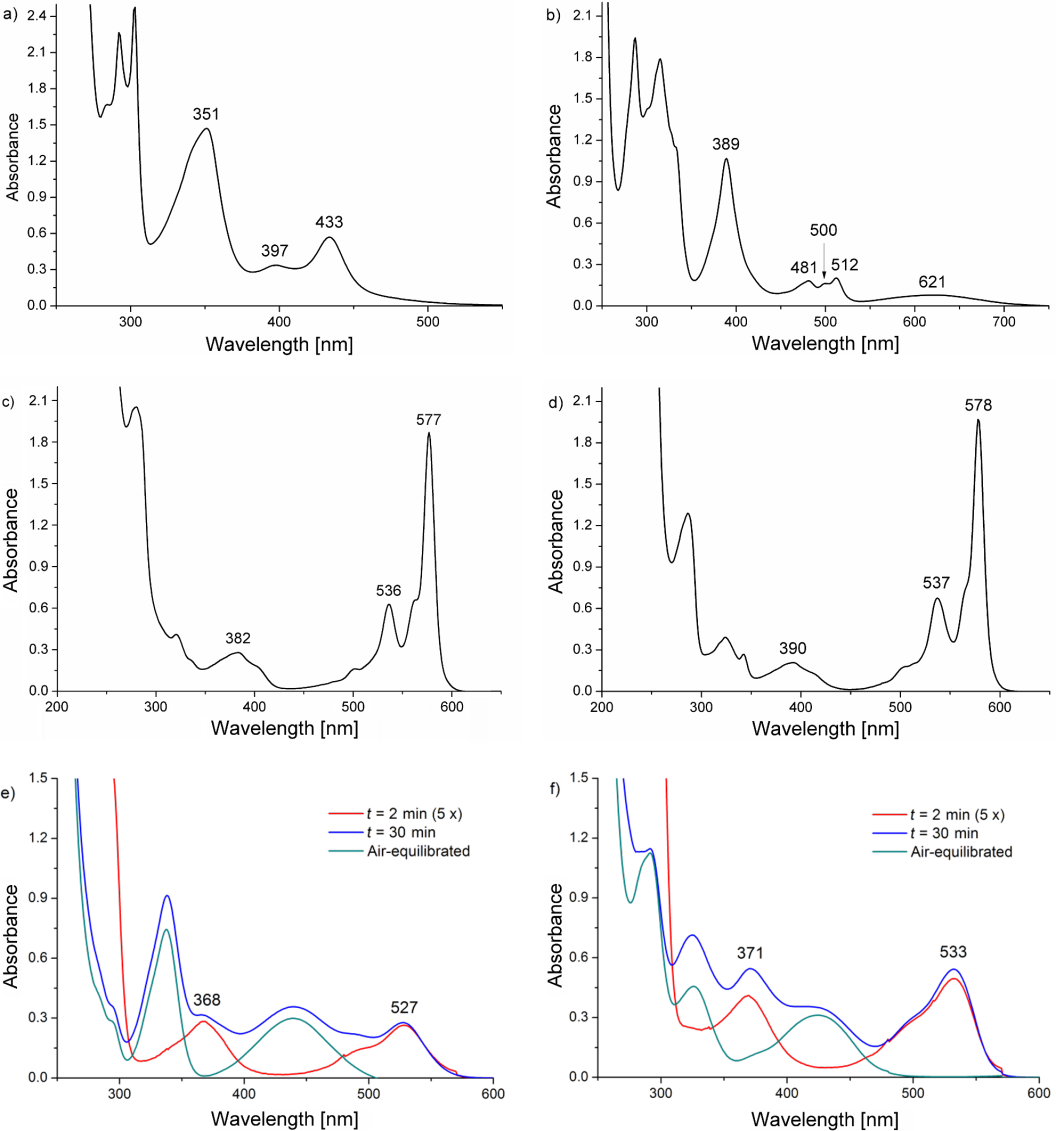
Absorption spectra of a freeze–pump–thaw deoxygenated MeCN solution irradiated at 313 nm of (a) 1,2,4-TrCB (5 × 10^−4^ M) in the presence of OXA (5.0 × 10^−2^ M), 20 min irradiation; (b) DCN (2 × 10^−4^ M) in the presence of OXA (5.0 × 10^−2^ M) and Bu_4_NH_2_PO_4_ (5 × 10^−3^ M), 60 min irradiation; (c) DCP (7.5 × 10^−5^ M) and OXA (5.0 × 10^−2^ M), 3 min irradiation; (d) DCP-Me (7.5 × 10^−5^ M) and OXA (5.0 × 10^−2^ M), 3 min irradiation; (e) ME (4 × 10^−4^ M) in the presence of OXA (5.0 × 10^−2^ M) after 2 (red) and 30 (blue) min irradiation and after air equilibration of the 30 min photolyzed solution (green); (f) PME (4 × 10^−4^ M) in the presence of OXA (5.0 × 10^−2^ M) after 2 (red) and 30 (blue) min irradiation and after air equilibration of the 30 min photolyzed solution (green).

The irradiation of a 5 × 10^−4^ M solution of 1,2,4-TrCB in the presence of OXA resulted in the development of a new absorption band, with sharp peaks at 351 and 433 nm and a broad shoulder around 400 nm, fully reversed on readmitting air. The signals were attributed to the 1,2,4-TrCB radical anion, to the best of our knowledge not previously characterized (see Figure 3a), on the basis of the analogy with TCB^•−^. This was not the case for 1,3,5-TrCB, where little if any of the radical anion [35,41–42] was formed and an irreversible modification of the spectrum occurred. Indeed none of the maxima known in the literature for this radical anion (532, 342 and 332 nm) developed [35].

Analogously, in the case of DCN the corresponding radical anion [40] was not detected, while a broad, non-oxygen-sensitive band at ca. 390 nm was observed. On the other hand, when the same irradiation was performed after the addition of Bu_4_NH_2_PO_4_ (5 × 10^−3^ M) the formation of pale green DCN^•−^ occurred with moderate efficiency (30% yield based on the ε of DCN^•−^ reported in the literature, 3578 L mol^−1^ cm^−1^ at 512 nm [40]; see Figure 3b). As for DCA, only a tiny amount of the radical anion [35] was generated (data not shown). A purple color and conspicuous bands developed in the case of phthalimides DCP and DCP-Me, with sharp peaks at 577–578 and 535–536 nm and a broad band at ca. 400 nm (see Figure 3c and Figure 3d), reversible with air. These were attributed to the corresponding radical anions, not previously characterized, but fitting with a computational prediction of 559 nm for the radical anion of the unsubstituted *N*-methylphthalimide [43]. Finally, with both of the aromatic esters investigated, the bands of the corresponding pink radical anions (ME, 368 and 527 nm [44]; PME, 371 and 533 nm, Bu_4_NH_2_PO_4_ added) were initially formed, but prolonged irradiation caused the formation of different absorption bands around 335 and 440 nm (see Figure 3e and Figure 3f). The former signals disappeared after air equilibration, while the latter persisted. Chromatographic analysis showed the formation of a strongly polar product (not identified).

## Discussion

Figure 2 and Figure 3 and comparison with the literature allow us to classify the behavior of the acceptors into three groups. Polycyanobenzenes (with the exception of 1,3,5-TrCB) and cyanophthalimides form conspicuously the radical anion. Where known (as in the case of TCB), the spectrum corresponds closely to that reported (with small differences due to the different medium), and the intensity of the signals observed suggests that the conversion is almost complete. The accumulation of such species up to quantitative yield is demonstrated in neat MeCN. This can be related to extensive localization of the charge at the electronegative atoms, which makes these radical anions nonbasic and not nucleophilic [19,23,38]. In the second group, DCN gives a much less than unitary amount (ca. 30%) of the radical anion, and DCA barely a trace. This is reasonably related to the lower charge delocalization on the nitrogen atoms with respect to the above benzonitriles. In accordance with this idea, the DCN radical anion has been shown to accumulate to a larger degree in the presence of salts that afford a better stabilization. Finally, the esters accumulate to a certain extent (ca. 30%), but at this level, within the time of the steady-state experiments, they begin to undergo some decomposition. These radical anions have been reported to undergo loss of the alkyl group [45], and this reasonably explains the observed irreversible decomposition. The notes above define the requirement for accumulation of radical anions: no chemical reaction (and this excludes esters) and sufficient stabilization by both aromatic substituent and medium.

**Scheme 2:**
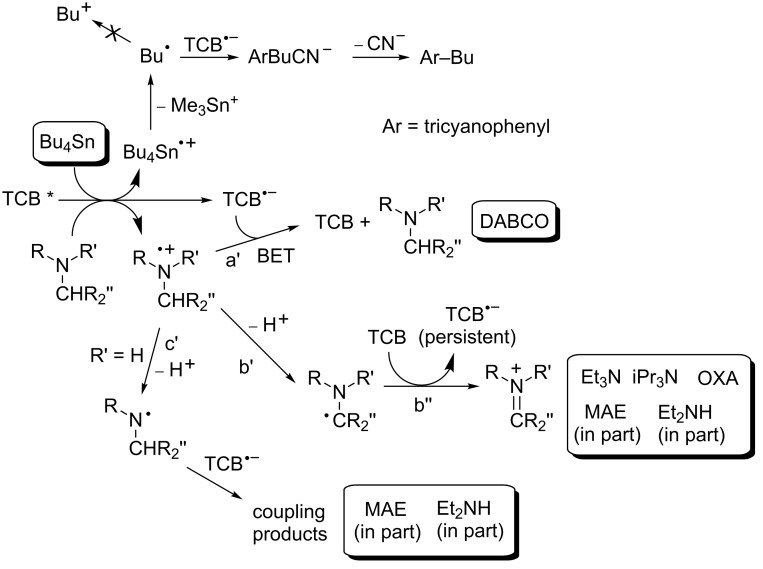
Mechanistic scheme.

On the other hand, the choice of the donor is equally decisive, because of the different reactivity of the corresponding radical cations. These may be classed again in three groups: those that undergo exclusively BET (Scheme 2, path a'), those that undergo an irreversible reaction not involving the radical anion (path b'), and those that react with it (path c').

**Figure 4:**
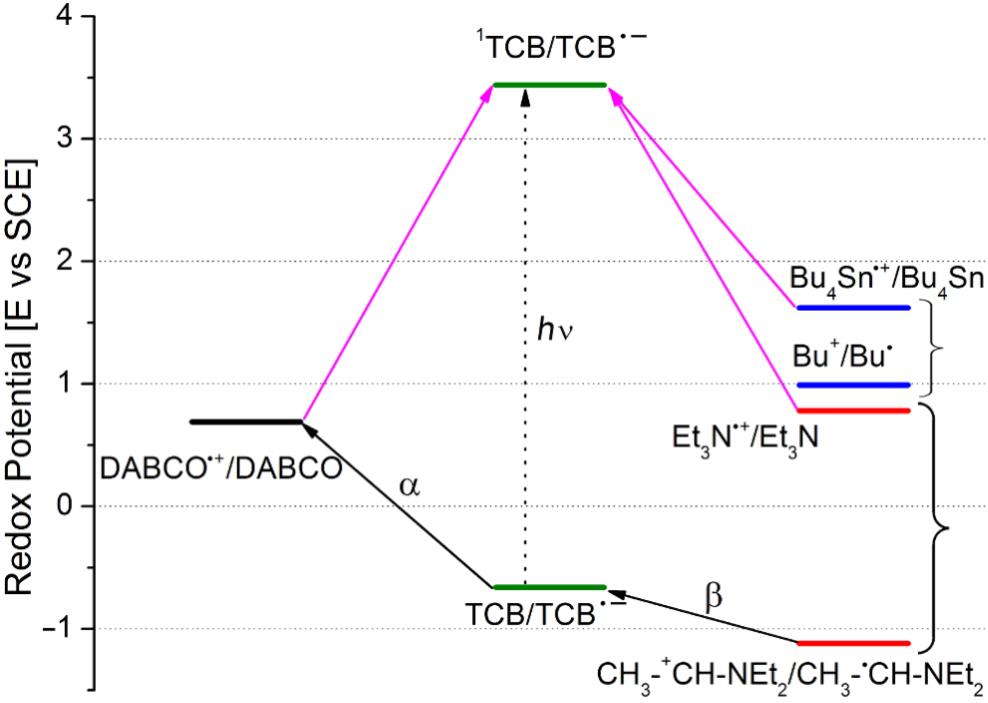
Thermodynamics of the redox processes discussed (solid arrows represent exergonic electron donation in the sense indicated). Thus, Et_3_N, Bu_4_Sn and DABCO all are oxidized by ^1^TCB (ET; purple arrows). Likewise BET from TCB^•−^ is always viable and is the only possibility for DABCO (path α). Et_3_N^•+^ cleaves and the radical generated is oxidized by TCB (path β), whereas this is not possible for the Bu^•^ radical formed by cleavage of the stannane (the potential of the Bu^+^/Bu^•^ couple is approximated here by that of the ethyl cation/ethyl radical couple [47]).

Thus, tertiary amines are known to undergo deprotonation from the α-position to produce a radical that is in turn easily oxidized to the iminium cation. In the present examples, such radicals are oxidized by the ground-state sensitizer (*E* = −1.12 V versus SCE for the α-aminoradical resulting from TEA, see path β in Figure 4 [46] and path b'' in Scheme 2). In this way, radical cations are destroyed, and a second equivalent of TCB^•−^ is generated and accumulated. The quantum yield is 2*k*_b'_ /(*k*_a'_ + *k*_b'_), although the measured value (0.15) is probably lower than the ideal value because of side processes in solution. Notice that, although TEA and OXA form TCB^•−^ at the same rate in rigorously deaerated solutions, only the latter donor allows accumulation in solutions that have been merely nitrogen flushed and capped. Separation of radical ions appears to be faster in OXA due to conformational factors, in particular the steric bulk of the methyl groups. The more efficiently formed radicals scoop away traces of oxygen still present under these conditions.

Likewise, in accordance with the role of amine radical cation deprotonation, is the fact that, of the two further tertiary amines, iPr_3_N causes a somewhat slower accumulation (the conformation of the radical cation is known to be less favorable for deprotonation) [48–50], and DABCO (for which deprotonation is impossible [51–52]) causes no detectable formation of TCB^•−^. Indeed, previous laser flash photolysis experiments had shown that DABCO formed the TCB radical anion on the nanosecond time scale just as the other tertiary amines [53]. However, no reaction able to eliminate radical cations was viable in this case, and thus BET predominated (path a' in Scheme 2 and path α in Figure 4).

Irreversible decomposition of TCB takes place to a small extent with DABCO, but it is much more conspicuous with secondary amines and the only path with the stannane. Radical cations of secondary amines are rather acidic (p*K*_a_ = 5.3 for Et_2_NH^•+^ [54]) and undergo both α-C–H and N–H deprotonation, the latter process being also kinetically favored. Furthermore, the aminyl radical has been reported to couple with cyanoaromatics in a nonreversible process as reported by Correa et al. [55]. This justifies the formation of a stable photoproduct non-reoxidized by oxygen (path c' in Scheme 2). As for the stannane, cleavage of the radical cation to give Bu_3_Sn^+^ and Bu^•^ is favored. The C-centered radical is not oxidized by TCB^•−^ (see Figure 4) but couples with it, resulting in efficient ipso-substitution of the nitrile (Φ 0.40) [18]. Thus, only path b' alone leads to the accumulation of the radical anion.

## Conclusion

The above experiments evidence the possibilities and the requirements for accumulating radical anions of electron-withdrawing substituted aromatics in neat organic solution. Apart from the spectroscopic interest, these data have a bearing on the mechanism of ET reactions such as those in Scheme 1 and Scheme 2. Aliphatic radical cations absorb poorly and are difficult to detect, but the conspicuous absorbance of the accompanying aromatic radical anions (the spectroscopic properties of which have been gathered in Table 1) gives most of the required information about the kinetic viability of the processes competing, offering through their bright colour a sort of litmus test for their evolution.

## Experimental

The photochemical acceptors TCB, DCA, as well as the donors triethylamine (Et_3_N), triisopropylamine (iPr_3_N), 1,4-diazabicyclo[2.2.2]octane (DABCO), diethylamine (Et_2_NH) and 2-(*N*-methylamino)ethanol (MAE) were commercially available. 1,3,5-TrCB [56], 1,2,4-TrCB [56], DCN [57], DCP [10], DCP-Me [10], ME [58], PME [11] and OXA [59] were obtained by known procedures. Since oxygen could interact with the photogenerated radical anions, resulting in a back oxidation [6], all of the experiments were carried out in freeze–pump–thaw deoxygenated solutions, except where otherwise noted. The examined solutions were irradiated on an optical bench equipped with a 150 W high-pressure mercury lamp, (λ_IRR_ = 313 nm). The electrochemical properties of TCB [8–9], DCN [8–9], DCA [8–9], DCP [10], DCP-ME [10], ME [45], PME [28], Et_3_N [60], iPr_3_N [61], DABCO [61] and Bu_4_Sn [62] were available in the literature. Electrochemical measurements (cyclic voltammetry) on the other compounds (Et_2_NH, MAE, OXA, 1,3,5-TrCB and 1,2,4-TrCB) were performed on 5 × 10^−2^ M solutions of the analyte in a three-electrode cell (volume 10 mL; *n*-Bu_4_NClO_4_ 0.1 M as the supporting electrolyte) with glassy carbon (diameter 2 mm) as the working electrode, Pt wire as the auxiliary electrode, and Ag/AgCl (3 M NaCl) as the reference electrode. Scan speed was 50 mV s^−1^. The potential range investigated was 0/+2.5 V for oxidation and 0/−2.5V for reduction processes. The electrochemical measurements were carried out by a BASi computer-controlled electrochemical analyzer. The potentials measured were then referred to SCE, applying the equation *E* (versus SCE) = *E* (versus Ag/AgCl; 3 M NaCl) − 35 mV. The redox potential of the excited state of compounds TCB [8–9], DCN [8–9], DCA [8–9], DCP [10] and DCP-ME [10] were taken from the literature, whereas the redox potentials of the excited state of 1,3,5-TrCB [21], 1,2,4-TrCB [21], ME [58] and PME [28] were determined by the Rehm–Weller equation [63].
